# Effect of Physician-Pharmacist Participation in the Management of Ambulatory Cancer Pain Through a Digital Health Platform: Randomized Controlled Trial

**DOI:** 10.2196/24555

**Published:** 2021-08-16

**Authors:** Lu Zhang, Howard L McLeod, Ke-Ke Liu, Wen-Hui Liu, Hang-Xing Huang, Ya-Min Huang, Shu-Sen Sun, Xiao-Ping Chen, Yao Chen, Fang-Zhou Liu, Jian Xiao

**Affiliations:** 1 Department of Pharmacy Xiangya Hospital Central South University Changsha China; 2 Institute for Rational and Safe Medication Practices National Clinical Research Center for Geriatric Disorders Xiangya Hospital, Central South University Changsha China; 3 Geriatric Oncology Consortium Tampa, FL United States; 4 Taneja College of Pharmacy University of South Florida Tampa, FL United States; 5 Department of Clinical Pharmacology Xiangya Hospital Central South University Changsha China; 6 Hunan Key Laboratory of Pharmacogenetics Institute of Clinical Pharmacology Central South University Changsha China; 7 Department of Pharmacy The Second Xiangya Hospital Central South University Changsha China; 8 College of Pharmacy and Health Sciences Western New England University Boston, MA United States; 9 College of Information Science and Engineering Hunan Normal University Changsha China

**Keywords:** cancer pain, self-management, ambulatory setting, digital health, physician-pharmacist

## Abstract

**Background:**

Self-management of ambulatory cancer pain is full of challenges. Motivated by the need for better pain management, we developed a WeChat-supported platform, Medication Housekeeper (MediHK), to enhance communication, optimize outcomes, and promote self-management in the home setting.

**Objective:**

We conducted a randomized controlled trial to assess whether the joint physician-pharmacist team through MediHK would provide better self-management of ambulatory patients with cancer pain.

**Methods:**

Patients were randomly assigned to either an intervention group or control group. During the 4-week study period, the pharmacist would send 24-hour pain diaries daily, adverse drug reaction (ADR) forms every 3 days, and the Brief Pain Inventory form every 15 days to patients in the intervention group via MediHK. If a patient needed a change in drug/dosage or treatment of an ADR after the comprehensive review, the pharmacist would propose pharmacological interventions to the attending physician, who was then responsible for prescribing or adjusting pain medications. If no adjustments were needed, the pharmacist provided appropriate targeted education based on knowledge deficits. Patients in the control group received conventional care and did not receive reminders to fill out the forms. However, if the control group patients filled out a form via MediHK, the pain management team would review and respond in the same way as for the intervention group. The primary outcomes included pain intensity and pain interference in daily life. Secondary outcomes included patient-reported outcome measures, medication adherence, ADRs, and rehospitalization rates.

**Results:**

A total of 100 patients were included, with 51 (51%) in the intervention group and 49 (49%) in the control group. The worst pain scores, least pain scores, and average pain scores in the intervention group and the control group were statistically different, with median values of 4 (IQR 3-7) vs 7 (IQR 6-8; *P*=.001), 1 (IQR 0-2) vs 2 (IQR 1-3; *P*=.02), and 2 (IQR 2-4) vs 4 (IQR 3-5; *P*=.001), respectively, at the end of the study. The pain interference on patients' general activity, mood, relationships with others, and interests was reduced, but the difference was not statistically significant compared with the control group (*Ps*=.10-.76). The medication adherence rate increased from 43% to 63% in the intervention group, compared with an increase of 33% to 51% in the control group (*P*<.001). The overall number of ADRs increased at 4 weeks, and more ADRs were monitored in the intervention group (*P*=.003). Rehospitalization rates were similar between the 2 groups.

**Conclusions:**

The joint physician-pharmacist team operating through MediHK improved pain management. This study supports the feasibility of integrating the internet into the self-management of cancer pain.

**Trial Registration:**

Chinese Clinical Trial Registry ChiCTR1900023075; https://www.chictr.org.cn/showproj.aspx?proj=36901

## Introduction

Pain is a common and challenging issue for cancer patients, affecting most at some stage of their disease [1]. A meta-analysis indicated that pain prevalence was 33% in patients after curative treatment, 64% in patients with advanced disease, and 59% in patients on anticancer therapy; approximately 35% of patients reported their pain as moderate to severe [2]. Inadequate pain management continues, with approximately one-third of cancer pain patients undertreated [3]. According to recent surveys, cancer pain management in China remains far from the ideal goal [4]. The barriers are multifactorial, including knowledge deficits, inadequate pain assessment, and misconceptions of pain from both patients and professionals [5]. Managing ambulatory patients is especially tough because of the complex environment, limited communication with health care providers, and difficulty managing their pain-medication regimens [6,7]. 

Both the World Health Organization and the European Society for Medical Oncology recommended that cancer pain patients should be active in their self-management of their pain. Patient-reported outcomes are increasingly used in routine ambulatory cancer care to guide clinical decisions, enhance communication, and improve symptom management [8]. Electronic patient-reported outcomes, supported by computer-adaptive testing technology, have shown potential in the era of big data [9]. Smartphones and apps such as WeChat (the largest social networking app in China), provide additional value to obtain knowledge and information, as well as making it possible for patients and health care providers to communicate electronically. Most patients are willing to self-report their symptoms via digital health apps. Several studies have reported on applications based on the eHealth model for the self-management of cancer pain [10,11].

This study established a physician-pharmacist collaboration team that participated in the self-management of ambulatory patients with cancer pain through a WeChat-supported platform: Medication Housekeeper (MediHK). We aimed to assess whether the joint physician-pharmacist team operating through MediHK would provide better self-management of ambulatory patients with cancer pain and optimize therapeutic outcomes.

## Methods

### WeChat-Supported Platform: MediHK Design

Patients were managed by MediHK, a WeChat-supported platform designed by the research team. An engineer from Hunan Normal University’s College of Information Science and Engineering provided technology support for building MediHK. MediHK has been patented by the National Intellectual Property Administration, People’s Republic of China (ZL 2015 1 0648320.2). MediHK contains 2 opening screens: (1) the patient interface (Multimedia Appendix 1) and (2) the medical interface (Multimedia Appendix 2). The former is for patients, and the latter is for the members of the pain management team, which consisted of physicians and pharmacists. The medical interface was designed to manage pain-related problems and provide consultation to patients in a timely fashion. Both interfaces included 3 modules: a user login module for inserting basic user demographics into MediHK; an e-consultation module for communicating between patients and medical providers; and an introductory module for MediHK education, which offers a quick response code for new users (Multimedia Appendix 3).

We conducted 3 rounds of consensus-building using Delphi methods [12] to determine patient-reported outcome measures (PROMs) that could be integrated into MediHK. The pharmacists sent messages to patients, as shown on the far-right side of Multimedia Appendix 2, and the patients would receive a reminder, as shown on the far-right side of Multimedia Appendix 1. Patients could consult on any questions about pain, and the pain management team would receive real-time WeChat messages and respond as soon as possible. The acceptable response time was generally within 2 hours. When patients needed a change in drug/dosage or treatment of an adverse drug reaction (ADR), pharmacists first reviewed the patients’ historical records through MediHK and, then, made recommendations and reminded the physician. In China, pharmacists have no right to prescribe. If the physician had conflicting opinions, an agreement would be reached through offline contact; then, the physician could adjust drug-therapy regimens. All treatment recommendations from the pharmacists and physicians were based on the guidelines of the European Society for Medical Oncology Standard diagnosis and treatment of cancer pain, the National Comprehensive Cancer Network, and the European Association for Palliative Care.

When the patient’s expression was not clear, there was no guarantee that the same physician or pharmacist would communicate back. However, since the previously submitted forms and consultation questions were available through MediHK, the new physician or pharmacist would review the patient’s history in all aspects. Patient information was protected by encryption. Note that all screenshots of the app include translations that have been added for clarity for this paper.

### PROMs and Forms That Integrated Into the MediHK

#### The Brief Pain Inventory

This study used the Brief Pain Inventory (BPI; Chinese version) to assess cancer pain, and it has been widely used for its good construct and concurrent validity [13]. It provides 2 main scores, which are “pain intensity” and “pain interference in daily life.” Pain intensity is based on the Numerical Rating Scale (NRS) and includes a 4-item sensory dimension: worst pain, least pain, average pain, and present pain. Each item is rated from 0 (“no pain”) to 10 (“very severe pain”). The “pain interference on daily life” score is a 7-item reactive dimension that includes general activity, mood, walking ability, daily work, relationships, sleep, and enjoyment of life; each item is rated from 0 (“no interference on daily life”) to 10 (“complete interference”).

#### Medication History and Adherence

We designed a list to record the medication history of ambulatory patients with cancer pain (Multimedia Appendix 4). The Morisky Medication Adherence Measure [14] was used to assess adherence to analgesics because of its excellent reliability and validity in the Chinese cancer pain population [15]. The Morisky Medication Adherence Measure focuses on the following medication-taking behaviors (Multimedia Appendix 5): forgetfulness, carelessness, and cessation of the drug regimen when feeling better or worse. The answers of “yes” or “no” for each item scored 0 and 1, respectively. The scores were divided into 3 categories: complete adherence (4), incomplete adherence (1-3), and nonadherence (0).

#### ADR Form

Many patients treated with opioids may experience adverse events. To comprehensively capture ADRs of patients outside of the hospital, we designed a form (Multimedia Appendix 6) with World Health Organization terminology for ADRs and classified ADRs into several symptoms.

#### Pain Diary for 24-Hour Pain

We designed a pain diary to capture patients’ daily pain in the home setting over time. The pain diary included 5 modules (Multimedia Appendix 7): (1) a pain score record, which combined the NRS, Face Pain Scale, and Verbal Rating Scale to assess pain accurately; (2) a form that included the time of administration as well as medication name and dosage for patients taking medication within 24 hours; (3) a module that recorded the specific duration, pain score, treatment status, and new pain location when the NRS was >4; (4) a module that recorded detailed pain information; and (5) a final module that gave suggestions to physicians or pharmacists based on patient feedback.

### Study Design and Participants

#### Study Overview

This was a 2-arm, randomized controlled clinical trial, and the study has been registered at Chictr.org ChiCTR1900023075; https://www.chictr.org.cn/showproj.aspx?proj=36901. Ambulatory patients with cancer pain in a tertiary hospital were included and assigned to either a control group (ie, joined in the MediHK only, no physician-pharmacist active intervention) or an intervention group (ie, MediHK platform plus physician-pharmacist intervention), with an allocation ratio of 1:1 using a random number table. Our pre-experiment included 72 patients who met the criteria for inclusion. The preliminary results showed that the average NRS of patients in the control group was 5.85 (SD 2.442). The average score of patients in the intervention group was expected to be <4. Assuming a type I error of 5%, a type II error of 20%, and considering the design of similar sample content, the sample size required for each group was calculated to be 37 patients. Allowing for 20% attrition, 100 patients (50 participants per group) were planned to be enrolled. The study was conducted in accordance with the Declaration of Helsinki and was approved by the ethics committee of Xiangya Hospital of Central South University (approval number 2017121139). All participants signed an informed consent form before participation.

#### Care of Patients in the Intervention Group

The pharmacist would send daily 24-hour pain diaries, ADR forms every 3 days, and the BPI form every 15 days via MediHK. The pharmacist would first review patient demographic information, check the form regarding pain intensity and interference in daily life, conduct medication therapy reviews, and review ADRs and medication adherence. If the patient needed a change in drug/dosage or treatment of an ADR after the comprehensive review, the pharmacist would propose pharmacological interventions to the attending physician. The physician was responsible for prescribing or adjusting pain medications. If no changes were needed, the pharmacist provided appropriate targeted education based on patient knowledge deficits.

#### Care of Patients in the Control Group

Patients in the control group received conventional care. Before the patient was discharged from the hospital, the pharmacist conducted detailed medication education. However, the control group patients did not receive a reminder to fill out the forms. If they filled out the form via MediHK, the pain management team would also review and respond the same way as for the intervention group.

### Inclusion and Exclusion Criteria

The inclusion criteria of participants included: (1) age ≥18 years; (2) diagnosis of malignant tumors by a pathological or cytological method; (3) pain that met the cancer pain diagnostic criteria according to National Comprehensive Cancer Network Guidelines and was moderate to severe (NRS ≥4); (4) ability of patients or their families to read Chinese and use WeChat; (5) a normal verbal ability and performance status; and (6) agreement to participate in the study and sign the informed consent form.

Exclusion criteria of participants were: (1) nonmalignant pain; (2) hepatic dysfunction (alanine aminotransferase ≥2.5×upper limits of normal [ULN], aspartate aminotransferase ≥2.5×ULN, or total bilirubin ≥1.5×ULN) or renal dysfunction (serum creatinine ≥2.5×ULN); (3) participation in other clinical trials; and (4) hospitalization during the 4-week trial period.

### Patient Enrollment and Intervention

#### Patient Enrollment

At the patients’ first visit to the ambulatory clinic, the physician provided a detailed consultation and, then, determined a treatment plan after discussion with the pharmacist; an account was created for eligible patients. After registration, the pharmacist demonstrated the use of each MediHK module to patients, including what information was collected in each form and how to fill it out and how to switch the interface to send a form or question. Even though the operation of MediHK was simple enough, the training process was approximately 10 minutes. The specific time depended on patient understanding, ability, and proficiency in WeChat. After training, patients were assigned to a pain management team and were required to complete PROMs and forms. Patients at home could contact their pain management team at any time through MediHK if they had any trouble with pain. The pain management team was required to complete standardized clinical pain management training and had at least 10 years of hospital work experience for clinical pharmaceuticals for cancer pain before performing pain-management work.

Patients were observed for 4 weeks. At week 4, the patients were required to complete and submit the PROMs through MediHK or report through phone calls within 1 day. Patients could continue to use MediHK after the completion of the study.

### Outcome Evaluation

The primary outcomes included pain intensity and pain interference in daily life. Secondary outcomes included PROMs, medication adherence, ADRs, and rehospitalization rates.

### Statistical Analysis

All data were analyzed using SPSS software (version 22.0; IBM Corp), and all charts were made by the graphing software GraphPad (version 8.0.2 (263); GraphPad Prism). For measurement data, the normality test adopted the Kolmogorov-Smirnov method. If normally distributed, the data were expressed as mean (SD), and the comparison between the 2 groups used 2 independent sample *t* tests. If not normally distributed, the data were expressed as the median (IQR), and the comparison between groups underwent a Mann-Whitney U test. Counting data were expressed as a frequency and percentage. A chi-square (c^2^) test or Fisher exact test was used for comparison between groups. We screened for factors affecting the pain intensity of outpatients with cancer pain by multivariate linear regression analysis (backward method, in=0.05, out=0.10). We defined *P*<.05 (test level=.05, two-tailed) as statistically significant.

## Results

### Principal Results

A total of 100 patients joined and completed this study, with 51 (51%) in the intervention group and 49 (49%) in the control group. Demographic information (ie, gender, age, height, and weight) and clinical information (ie, diagnosis, pain type, and site of pain) of the 2 groups were not statistically different, nor was the intensity, pain interference, and adherence to pain medications at baseline (*Ps*>.05; Table 1).

**Table 1:** Baseline characteristics of patients.

Variable		Intervention group (n=51)	Control group (n=49)	*P* value (statistical test)
**Demographic information**				
	Male, n (%)	38 (75)	34 (69)	.57 (χ^2^=0.325)
	Age (years), mean (SD)	54.6 (14.0)	58.7 (14.8)	.16
	Height (cm), median (IQR)	166.0 (160.0-170.0)	166.0 (160.0-169.5)	.29
	Weight (kg), median (IQR)	55.0 (47.7-65.0)	55.0 (50.0-60.0)	.52
**Diagnosis, n (%)**				.33 (χ^2^=4.653)
	Lung cancer	16 (31)	24 (49)	
	Gastrointestinal cancer	19 (37)	14 (29)	
	Head and neck cancer	6 (12)	2 (4)	
	Breast cancer	2 (4)	1 (2)	
	Other	8 (16)	8 (16)	
**Pain site, n (%)**				.16 (χ^2^=7.986)
	≥2 sites	26 (51)	30 (61)	
	Chest and abdomen	6 (12)	8 (16)	
	Head and neck	6 (12)	5 (10)	
	Back	6 (12)	5 (10)	
	Limbs	5 (10)	0 (0)	
	Other sites	2 (4)	1 (2)	
**Pain type, n (%)**				.08 (χ^2^=6.801)
	Mixed pain	20 (39)	21 (43)	
	Visceral pain	27 (53)	16 (21)	
	Neuropathic pain	3 (6)	9 (18)	
	Body pain	1 (2)	3 (6)	
**Pain intensity, median (IQR)^a^**				
	Worst pain	7 (5-8)	7 (6-9)	.16
	Least pain	2 (1-3)	2 (1-3)	.26
	Average pain	4 (2-6)	4 (3-6)	.33
	Present pain	2 (1-4)	3 (1-4)	.17
**Pain interference, median (IQR)^a^**				
	General activity	7 (4-10)	6 (3-8)	.31
	Mood	5 (2-8)	5 (4-7)	.43
	Walking ability	8 (2-10)	5 (2-9)	.27
	Daily work	9 (4-10)	7 (4-9)	.10
	Relationships	3 (2-6)	3 (2-6)	.94
	Sleep	7 (4-9)	6 (5-9)	.88
	Enjoyment of life	5 (2-7)	6 (2-8)	.61
**Baseline adherence, n (%)**				.10 (χ^2^=2.784)
	Nonadherence	3 (6)	8 (16)	
	Incomplete adherence	26 (51)	25 (51)	
	Complete adherence	22 (43)	16 (33)	

^a^These measures represent the baseline characteristics based on the Brief Pain Inventory.

### BPI Outcomes

Pain intensity in the intervention group was significantly reduced compared with the control group. The worst pain scores, least pain scores, and average pain scores in the 2 groups were statistically different, with median values of 4 (IQR 3-7) vs 7 (IQR 5-8; *P*=.001), 1 (IQR 0-2) vs 2 (IQR 1-3; *P*=.02), and 2 (IQR 2-4) vs 4 (IQR 3-5; *P*=.001), respectively, favoring the intervention group. The difference in the present pain score of the 2 groups was not statistically significant (*P*=.23). However, the score of the intervention group was numerically lower than that of the control group (Table 2).

**Table 2:** Brief Pain Inventory outcomes at week 4.

BPI^a^ item		Intervention group (n=51), median (IQR)	Control group (n=49), median (IQR)	*P* value
**Pain intensity**				
	Worst pain	4 (3-7)	7 (5-8)	.001
	Least pain	1 (0-2)	2 (1-3)	.02
	Average pain	2 (2-4)	4 (3-5)	.001
	Present pain	1 (0-3)	2 (0-4)	.23
**Pain interference**				
	General activity	7 (4-8)	6 (3-8)	.76
	Mood	3 (1-6)	4 (2-6)	.58
	Walking ability	7 (4-10)	7 (3-8)	.32
	Daily work	8 (6-10)	8 (6-9)	.15
	Relationships	2 (1-4)	3 (1-5)	.64
	Sleep	4 (1-7)	7 (3-8)	.10
	Enjoyment of life	4 (2-7)	5 (2-8)	.43

^a^BPI: Brief Pain Inventory.

### PROM Submission Through MediHK

The number of forms submitted by the intervention group patients was much higher than that of the control group (710 vs 95), with an average of 4.64 forms per person per day vs 0.06 forms per person per day, respectively. The most common forms submitted by the control group were the BPI (53/95, 56%), pain diary (17/85, 18%), and medication list (15/95, 16%; Table 3). Even though the control group patients did not receive reminders to fill out the forms, they still actively contacted the pain management team through MediHK due to uncontrollable pain intensity, interference in daily life, or severe ADRs.

**Table 3:** Number of PROMs submitted by the 2 groups.

Form	Intervention group (n=710), n (%)	Control group (n=95), n (%)	*P* value (χ^2^=153.236)
Pain diary	495 (69.7)	17 (18)	<.001
ADR^a^ form	87 (12.2)	7 (7)	<.001
BPI^b^	83 (11.7)	53 (56)	<.001
Medication list	31 (4.4)	15 (16)	<.001
MMAM^c^	14 (2.0)	3 (3)	<.001

^a^ADR: adverse drug reactions.

^b^BPI: Brief Pain Inventory.

^c^MMAM: Morisky Medication Adherence Measure.

### Medication Adherence

**Figure 1:**
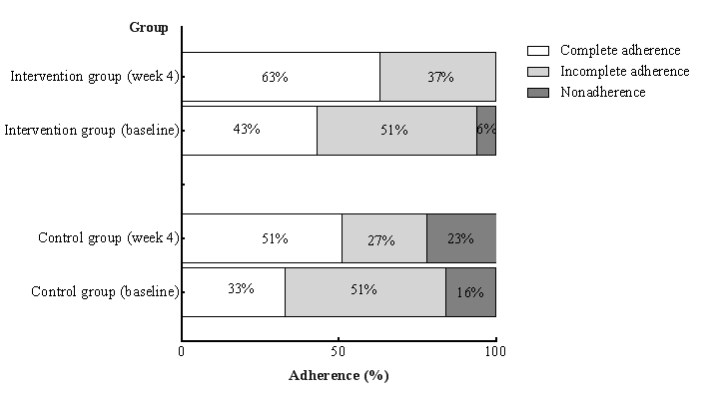
The adherence rate of the 2 groups at baseline and at week 4.

The complete adherence rate in the intervention group increased from 43% (22/51) to 63% (32/51), while that of the control group increased from 33% (16/49) to 51% (25/49; χ^2^=12.864; *P*<.001; Figure 1).

### Adverse Drug Reactions

The overall incidence of ADRs was 36% (36/100) across the 2 groups at baseline and increased to 56% (56/100) at week 4. ADR incidence in the intervention group was significantly higher than in the control group (χ^2^=8.990; *P*=.003). In addition, 3 cases of intestinal obstruction and 2 cases of delirium were observed in the intervention group. Table 4 shows the distribution of ADRs between groups.

**Table 4:** Adverse drug reactions between groups over 4 weeks.

Variable		Baseline			Week 4		
		Intervention group (n=51), n (%)	Control group (n=49), n (%)	*P* value (statistical test)	Intervention group (n=51), n (%)	Control group (n=49), n (%)	*P* value (statistical test)
Patients with ADR^a^		24 (47)	12 (25)	.02 (χ^2^=5.525)	36 (71)	20 (41)	.003 (χ^2^=8.990)
**ADR type**							
	Constipation	18 (35)	6 (12)	—^b^	29 (57)	14 (29)	—
	Nausea and vomiting	13 (26)	6 (12)	—	17 (33)	11 (22)	—
	Drowsiness	4 (8)	3 (6)	—	9 (18)	3 (6)	—
	Dizziness	6 (12)	1 (2)	—	9 (18)	3 (6)	—
	Pruritus	2 (4)	—	—	3 (6)	—	—
	Urinary retention	2 (4)	—	—	5 (10)	—	—
	Ileus	—	—	—	3 (6)	—	—
	Delirium	—	—	—	2 (2)	—	—

^a^ADR: adverse drug reaction.

^b^Not available.

### Rehospitalization Rates During the 4 Weeks

The 2 groups had a similar rehospitalization rates within the 4-week trial. There was no significant difference between the 2 groups within 4 weeks (χ^2^=0.010; *P*=.92).

### Analysis of Pain Factors

We introduced possible factors that could contribute to pain intensity for each pain item in a multivariate linear regression analysis. The physician-pharmacist intervention through MediHK was an independent influencing factor for the most severe pain (β=–1.413; *P*=.005; Multimedia Appendix 8) and average pain (β=–1.154; *P*=.003; Multimedia Appendix 9). Aside from medication adherence, the intervention was significantly related to the least pain（β=–.701; *P*=.02; Multimedia Appendix 10). No factors significantly influenced the present pain (gender β=1.078, *P*=.16; age β=.018, *P*=.26; height β=.063, *P*=.18; weight β=–.017, *P*=.46; adherence β=–.282, *P*=.40; intervention β=–.598, *P*=.17; Multimedia Appendix 11).

## Discussion

### Principal Results

The self-management of cancer pain is full of challenges, especially, for ambulatory patients. Approximately 70% to 90% of cancer patients can relieve pain adequately when carefully following the treatment guidelines. Now more fully developed, digital health helps to achieve good pain management in daily practice for ambulatory patients with cancer pain, particularly, in remote areas of China [6,11]. Patients’ various demands in supporting self-management help encourage the development of a multimodal web application [16,17].

This study included a joint physician-pharmacist team that managed ambulatory patients with cancer pain through a WeChat-supported platform, MediHK, with promising results. Even if the control group did not receive a reminder to fill out the forms, patients in this group actively contacted the pain management team through MediHK to determine whether the medication plan needed to be adjusted due to either uncontrollable pain intensity, interference on daily life, or severe ADRs. The results revealed the patients’ need to contact the professional team via MediHK for better pain management. The patients in the intervention group reported more ADRs compared with control group patients, primarily, because there were more reports obtained from intervention group patients. More ADRs were not in conflict with improving pain. For example, the pain management team added new drugs for pain treatment, which may have caused some ADRs, but most of them were tolerated after a few days and monitored closely by the pharmacist.

### Comparison With Prior Work

Yang et al [18] developed an app named Pain Guard for better pain management of discharged patients. Its functions were similar to MediHK, such as self-evaluation, real-time medication consultation, and record-keeping. The differences were that, for MediHK, we combined the NRS, Face Pain Scale, and Verbal Rating Scale to assess pain intensity accurately, while Pain Guard had only the traditional scale, NRS. We designed the module to record more medication-related details from patients, including drug name, dose, frequency, initial stop time, pain relief after medication, and adverse reactions. In addition, the physician or pharmacist could send forms embedded in MediHK, such as the ADR or adherence assessment form, to patients according to their status. These functions were unavailable in the Pain Guard. Scriven et al [19] used the BPI to evaluate patients’ pain while participating in the multisite telehealth group model. They found positive changes on the interference scale at the individual level (14% of patients) but no change at the group level. Another study offered standardized education and telemonitored for pain improvement, and BPI results indicated that, at 1 week, there were improvements in both the worst pain (from 7.3 to 5.7; *P*<.01) and average pain (from 4.6 to 3.8; *P*<.01) [20]. However, the portion of average pain rated ≥4 did not improve significantly because of the short study period [20]. Compared with telehealth, MediHK was more capable of real-time feedback.

Furthermore, we received more positive results because of our 4-week observation time. One study evaluated the effectiveness of pain management of a mobile phone app. Results showed that the pain relief rate was significantly different between the trial and the control groups (median 50, IQR 45-63 and median 0, IQR 0-25) [18]. Similarly, Sun et al [21] found a significant difference in the average pain score through an intelligent pain management system (mean 2.5, SD 0.42 vs mean 2.8, SD 0.47; *P*<.01). These findings support our vision of making full use of prescient and promising internet platforms to manage cancer pain. In another study with an internet application consisting of a pain diary and a pain education and consultation module, the present pain intensity and the worst pain intensity of patients in the intervention group were significantly reduced within 6 weeks [6]. MediHK found similar positive results and included more details in the pain diary module, such as recording all patient medication information and pain self-assessments at all times. It is worth mentioning that these studies were based on the NRS for pain assessment. However, MediHK also embedded the BPI form to consider pain itself and the interference it caused. During the 4 weeks, the worst pain intensity, least pain intensity, and average pain intensity of intervention group patients significantly reduced, with an average decrease of 1-2 points. In terms of pain interference, the impact of pain on patients' general activity, mood, relationships with others, and interests reduced. However, the difference was not statistically significant when compared with the control group. Intervention group patients showed significant improvements in adherence after 4 weeks, resulting from active interventions, raised awareness of patients, and real-time monitoring of ADRs, which was more accessible in the home setting through MediHK.

Regrettably, we did not record the impact of education status and age in keeping medical records. Patients who had never received education may take longer to keep records. However, since the included patients or their families were all able to use WeChat proficiently, we believed that MediHK was feasible; for patients who were too old or unable to record, family members or caregivers would help to send the form. In total, we accounted for the universal applicability of MediHK when we developed it to ensure easy operation. The only complicated step was the switch between the interface. However, this was emphasized when training in the outpatient clinic.

Knowledge deficits, inadequate pain assessment, misconceptions of pain, complex environments, and infrequent communication with health care providers are barriers in pain management. A joint physician-pharmacist team operating through a digital health platform can improve it. The cancer patient pain assessment was complicated. It is necessary to select quantification tools and assess the cause, location, quality, and relieving or aggravating factors of the pain comprehensively. The time that physicians spend on each patient is limited, and it is difficult to provide long-term and continuous monitoring. The digital platform can better solve these problems. The platform trains patients to record their pain conditions in a more standardized and targeted manner. During clinical encounters, clinicians can spend more time addressing patients’ concerns in a meaningful way, rather than running through checklists of questions [22]. In addition, this platform promotes patient self-management. It allows patients to pay attention to the daily changes in pain and offers a digital tool to seek out the help of a professional team when suffering from an intractable pain or serious ADRs.

It is essential to consider the clinical workflow, security and liability, and the time-cost. We conducted a preliminary investigation and consideration in the early stage and carried out several rounds of related process optimizations and software improvements. In addition, when patients first visited the clinic, we would state that their physicians and pharmacists would provide the home services via the platform, and patients trusted this service. Finally, patients signed an exemption agreement and informed consent to ensure medical safety before using the platform. The satisfaction of patients and medical workers on such digital health platforms matters. One study designed a module in a mobile app to survey overall satisfaction, and the questionnaire was completed by participants at the end of the study [18]. Another study also assessed patient satisfaction about the convenience and helpfulness of using mobile systems, receiving technical software support, receiving consultant and training courses, and prompt responses for help; the results indicated that patients had a high level of satisfaction toward these kinds of digital tools [21]. Our preliminary idea was to evaluate satisfaction by embedding a questionnaire. For patients, this included assessing the pattern of the platform and the pain management team’s joint management, the content of medication education, the acceptability of response time, and the overall services. For the pain management team, this included evaluating the ease of operation of the platform, the acceptability of clinical workflow interference, and working-time costs. The questionnaire could contain an open-ended question, in which both patients and the pain management team are encouraged to provide suggestions regarding improvements to MediHK.

### Study Strengths

There were some strengths of this study. First, this study was a real-world randomized controlled trial conducted in a large ambulatory clinic of a tertiary hospital. All patients were clinically recruited and randomly assigned. The integration of PROMs has not been a feature of other eHealth and (web) application–related studies, allowing this digital health study to help advance this field. In addition, real‐time reporting can facilitate just‐in‐time interventions based on an individual's current circumstance or environment. This study achieved real-time communication between ambulatory patients with cancer pain and health care providers through MediHK, extending medical services to ambulatory patients as a pathway for the self-management in home settings.

### Study Limitations

The study had the several limitations. First, this study had abnormally high participation, which will not necessarily reflect what would happen when patients use the platform independently, because pharmacists would send daily notifications. Second, it was prospectively powered and conducted in a randomized manner, but inevitable confounding factors can exist in the real world. Multivariate linear regression can only explain a small part of the influence of different pain intensity types. Third, since the study was conducted in a single tertiary hospital, applying this approach in other clinical settings may require some individualization to meet specific needs. Fourth, the observation time of only 4 weeks limited the long-term application of the results. Fifth, this study lacked further assessment about buy-in from both patients and the pain management team.

### Conclusions

The joint physician-pharmacist team operating through MediHK enhanced communication, optimized outcomes, and promoted self-management of patients in home settings. This study supports the feasibility of integrating the internet into patient self-management of cancer pain. In the future, it will be necessary to enlarge the sample size to further explore the long-term effects of this method on the self-management of ambulatory patients with cancer pain.

## Appendix

Multimedia Appendix 1 Screenshots of the patient interface, including registration, homepage, and reminders.

Multimedia Appendix 2 Screenshots of the medical interface, including reminders, review, homepage, and message-sending.

Multimedia Appendix 3 Screenshots of the introduction of the MediHK.

Multimedia Appendix 4 Medication history list. English translation shown on right.

Multimedia Appendix 5 Adherence Assessment Measurement. English translation shown on right.

Multimedia Appendix 6 Adverse Reaction Form. English translation shown on right.

Multimedia Appendix 7 Pain diary. English translation shown on right.

Multimedia Appendix 8 The independent factors influencing worst pain intensity.

Multimedia Appendix 9 The independent factors influencing average pain intensity.

Multimedia Appendix 10 The independent factors influencing least pain intensity.

Multimedia Appendix 11 The independent factors influencing present pain intensity.

Multimedia Appendix 12 CONSORT-eHEALTH checklist (V 1.6.1).

## Abbreviations

ADR: adverse drug reaction
BPI: Brief Pain Inventory
MediHK: Medication Housekeeper
NRS: Numerical Rating Scale
PROM: patient-reported outcome measures
ULN: upper limits of normal
