# Correction: Effect of Physician-Pharmacist Participation in the Management of Ambulatory Cancer Pain Through a Digital Health Platform: Randomized Controlled Trial

**DOI:** 10.2196/33223

**Published:** 2021-09-13

**Authors:** Lu Zhang, Howard L McLeod, Ke-Ke Liu, Wen-Hui Liu, Hang-Xing Huang, Ya-Min Huang, Shu-Sen Sun, Xiao-Ping Chen, Yao Chen, Fang-Zhou Liu, Jian Xiao

**Affiliations:** 1 Department of Pharmacy Xiangya Hospital Central South University Changsha China; 2 Institute for Rational and Safe Medication Practices, National Clinical Research Center for Geriatric Disorders, Xiangya Hospital, Central South University Changsha China; 3 Geriatric Oncology Consortium Tampa, FL United States; 4 Taneja College of Pharmacy, University of South Florida Tampa, FL United States; 5 Department of Clinical Pharmacology, Xiangya Hospital, Central South University Changsha China; 6 Hunan Key Laboratory of Pharmacogenetics, Institute of Clinical Pharmacology, Central South University Changsha China; 7 Department of Pharmacy, The Second Xiangya Hospital, Central South University Changsha China; 8 College of Pharmacy and Health Sciences, Western New England University Boston, MA United States; 9 College of Information Science and Engineering, Hunan Normal University Changsha China

In “Effect of Physician-Pharmacist Participation in the Management of Ambulatory Cancer Pain Through a Digital Health Platform: Randomized Controlled Trial” (JMIR Mhealth Uhealth 2021;9(8):e24555), one error was noted.

In the originally published article, the name of Corresponding Author “Jian Xiao” was formatted incorrectly as “Xiao Jian.” The original order of authors was listed as follows:

Lu Zhang, Howard L McLeod, Ke-Ke Liu, Wen-Hui Liu, Hang-Xing Huang, Ya-Min Huang, Shu-Sen Sun, Xiao-Ping Chen, Yao Chen, Fang-Zhou Liu, Xiao Jian

This has been corrected to:

Lu Zhang, Howard L McLeod, Ke-Ke Liu, Wen-Hui Liu, Hang-Xing Huang, Ya-Min Huang, Shu-Sen Sun, Xiao-Ping Chen, Yao Chen, Fang-Zhou Liu, Jian Xiao

The correction will appear in the online version of the paper on the JMIR Publications website on September 13, 2021, together with the publication of this correction notice. Because this was made after submission to PubMed, PubMed Central, and other full-text repositories, the corrected article has also been resubmitted to those repositories.

