# Supplementary figures and images for: Effect of Physician-Pharmacist Participation in the Management of Ambulatory Cancer Pain Through a Digital Health Platform: Randomized Controlled Trial
Source: JMIR Mhealth Uhealth. 2021 Aug 16;9(8):e24555. doi: 10.2196/24555 (PMC8406114; doi:10.2196/24555)

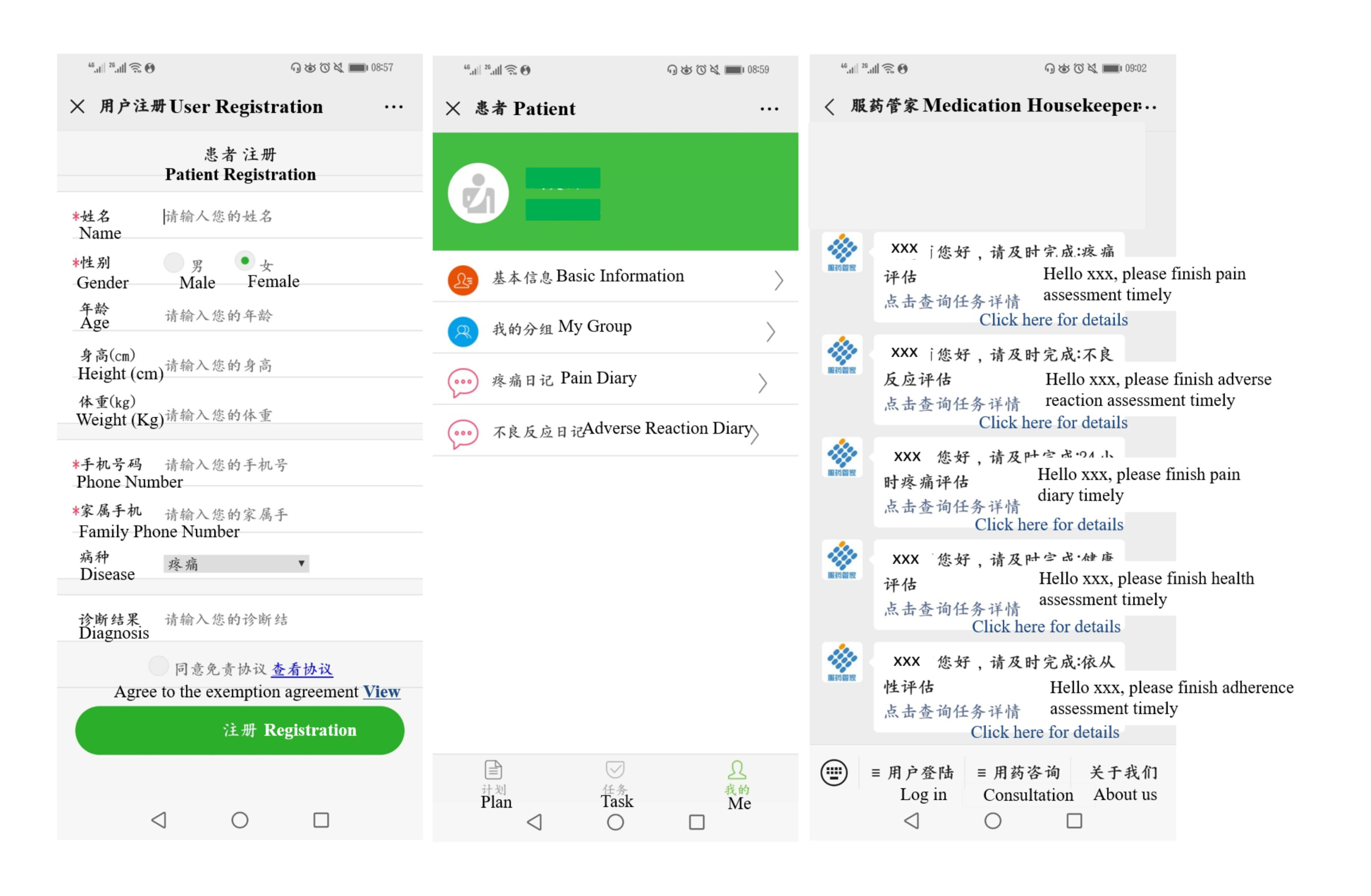

Supplement: Multimedia Appendix 1 [file mhealth_v9i8e24555_app1.png]

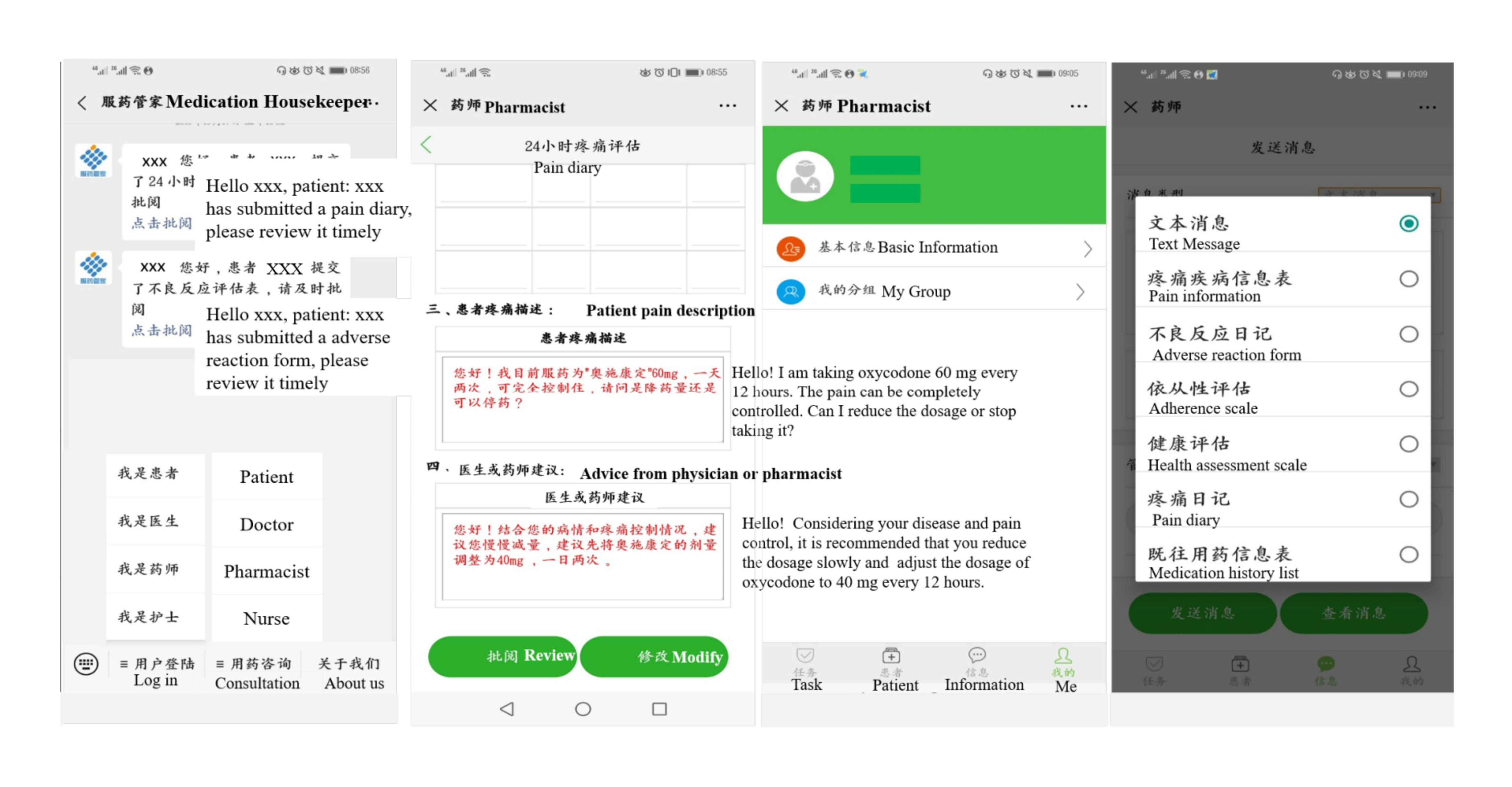

Supplement: Multimedia Appendix 2 [file mhealth_v9i8e24555_app2.png]

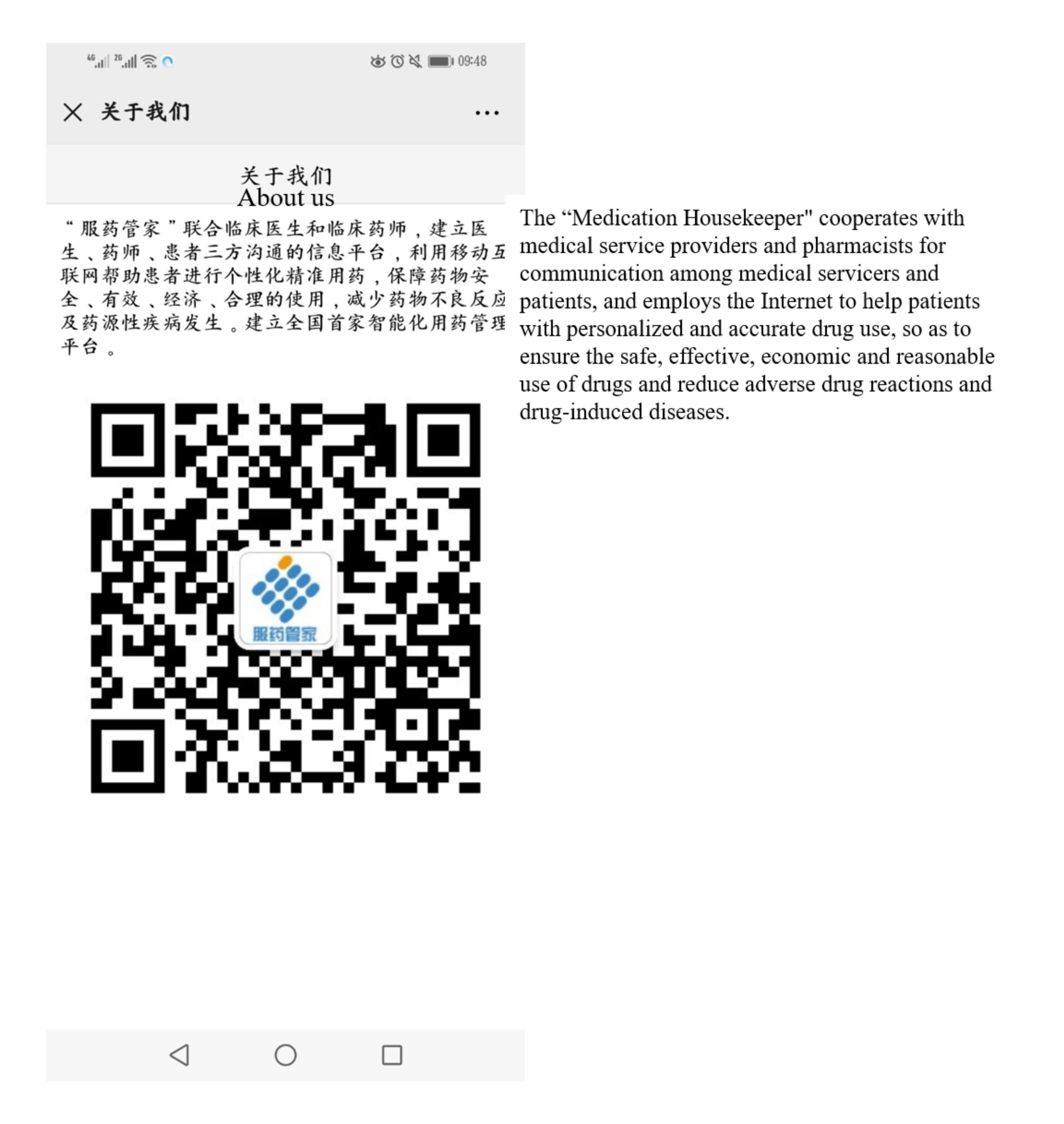

Supplement: Multimedia Appendix 3 [file mhealth_v9i8e24555_app3.png]

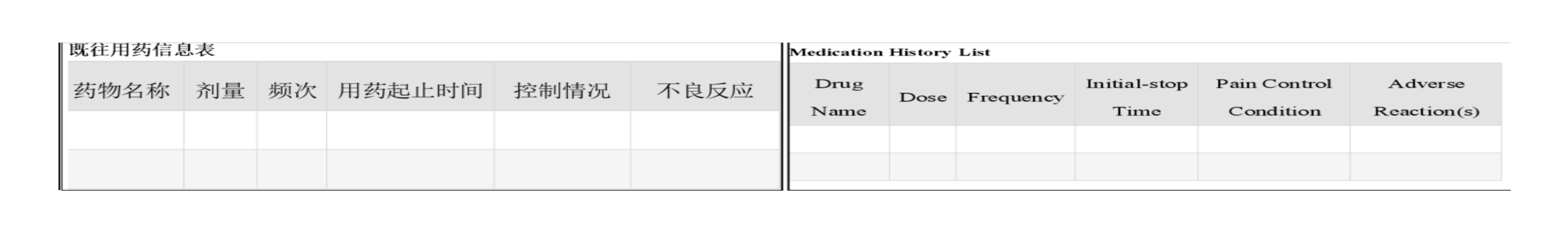

Supplement: Multimedia Appendix 4 [file mhealth_v9i8e24555_app4.png]

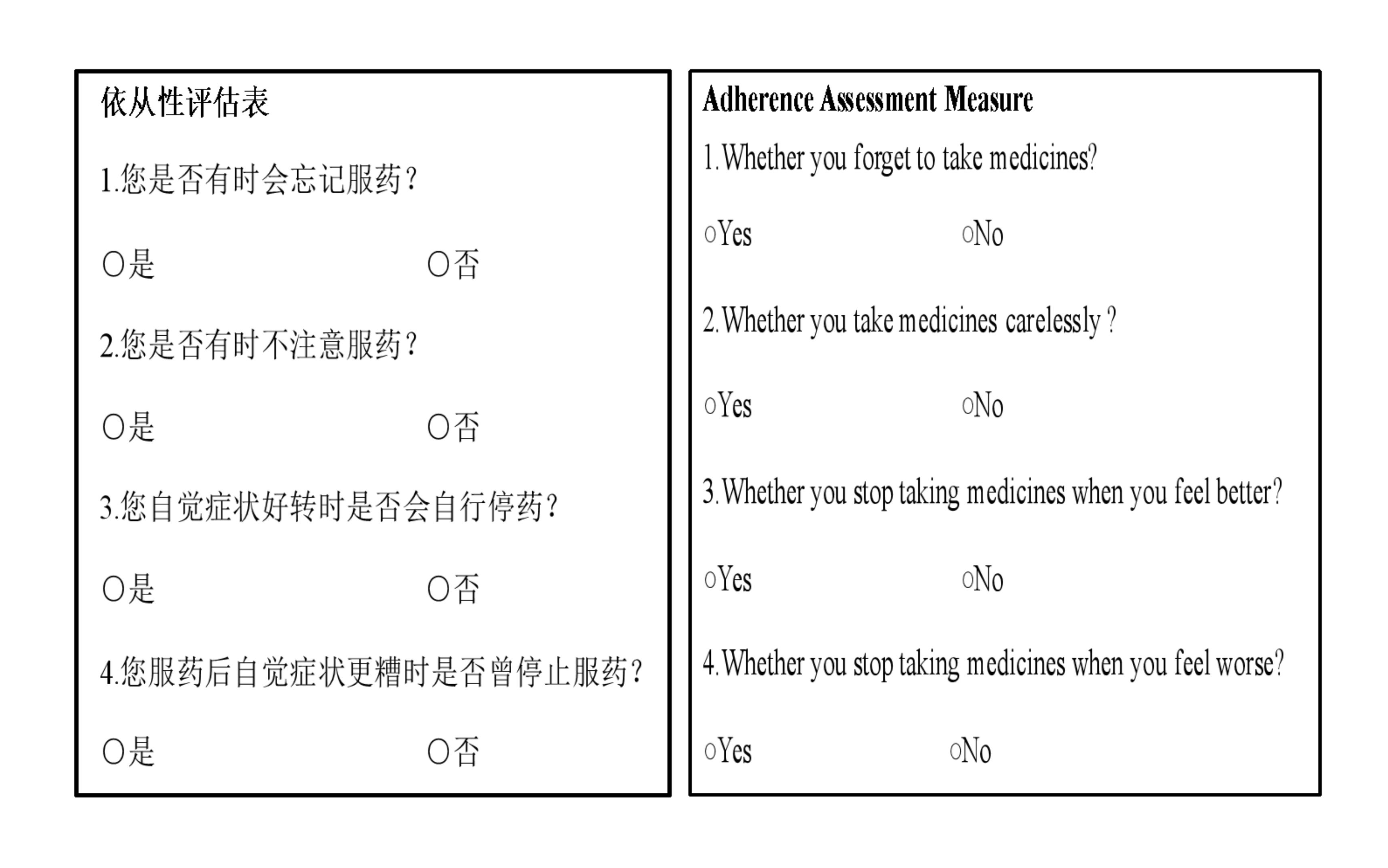

Supplement: Multimedia Appendix 5 [file mhealth_v9i8e24555_app5.png]

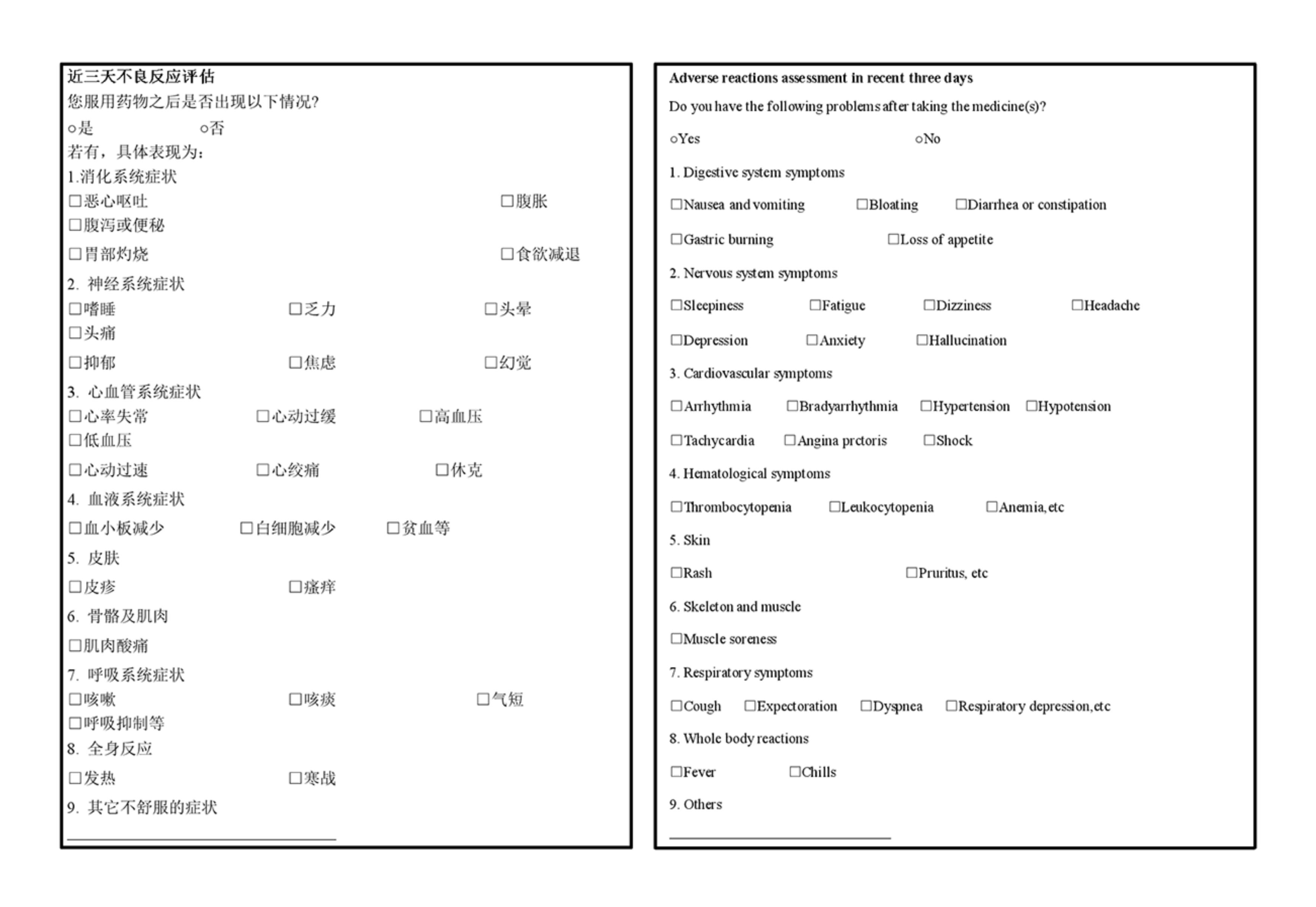

Supplement: Multimedia Appendix 6 [file mhealth_v9i8e24555_app6.png]

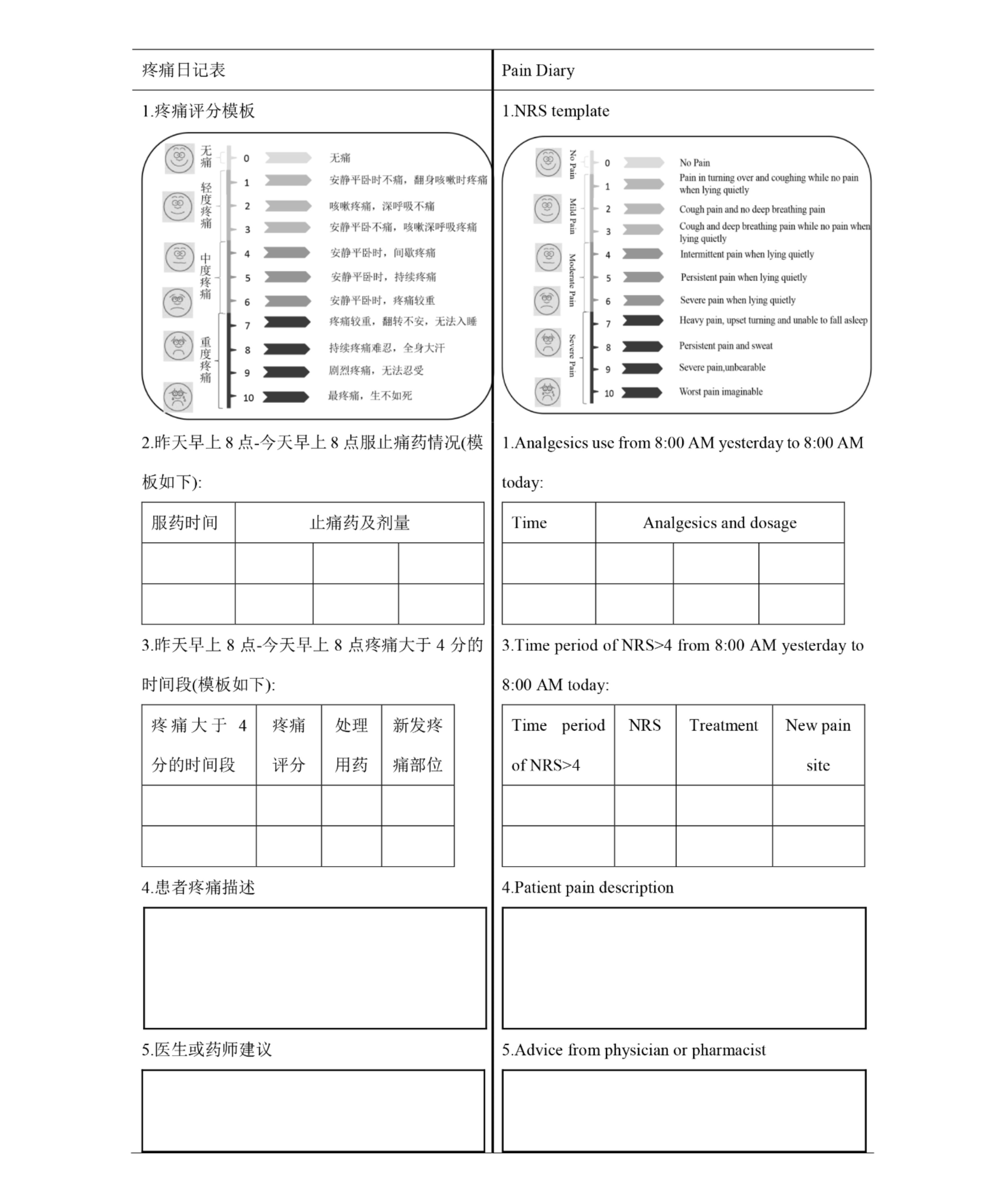

Supplement: Multimedia Appendix 7 [file mhealth_v9i8e24555_app7.png]
