# Supplementary material for: Effect of Physician-Pharmacist Participation in the Management of Ambulatory Cancer Pain Through a Digital Health Platform: Randomized Controlled Trial
Source: JMIR Mhealth Uhealth. 2021 Aug 16;9(8):e24555. doi: 10.2196/24555 (PMC8406114; doi:10.2196/24555)
Supplement: Multimedia Appendix 8 [file mhealth_v9i8e24555_app8.doc]

**Multimedia Appendix 8.** The independent factors influencing worst pain intensity.

| Parameters | β | SE | β’ | *P* - value | 95% CI of β | | R^2^ |
| --- | --- | --- | --- | --- | --- | --- | --- |
|  |  |  |  |  | Lower limit | Upper limit |  |
| Constant | -5.680 | 10.127 |  | 0.58 | -25.812 | 14.452 | 0.181 |
| Gender | 0.802 | 0.859 | 0.154 | 0.35 | -0.906 | 2.510 |  |
| Age | 0.018 | 0.019 | 0.113 | 0.33 | -0.019 | 0.056 |  |
| Height | 0.069 | 0.054 | 0.219 | 0.20 | -0.037 | 0.176 |  |
| Weight | -0.033 | 0.026 | -0.139 | 0.22 | -0.086 | 0.020 |  |
| Adherence | -0.200 | 0.376 | -0.058 | 0.60 | -0.947 | 0.548 |  |
| Intervention | -1.413 | 0.492 | -0.302 | **0.005** | -2.390 | -0.435 |  |
