# Supplementary material for: Effect of Physician-Pharmacist Participation in the Management of Ambulatory Cancer Pain Through a Digital Health Platform: Randomized Controlled Trial
Source: JMIR Mhealth Uhealth. 2021 Aug 16;9(8):e24555. doi: 10.2196/24555 (PMC8406114; doi:10.2196/24555)
Supplement: Multimedia Appendix 9 [file mhealth_v9i8e24555_app9.doc]

**Multimedia Appendix 9.** The independent factors influencing average pain intensity.

| Parameters | β | SE | β’ | *P* - value | 95% CI of β | | R^2^ |
| --- | --- | --- | --- | --- | --- | --- | --- |
|  |  |  |  |  | Lower limit | Upper limit |  |
| Constant | -5.748 | 7.723 |  | 0.46 | -21.100 | 9.604 | 0.161 |
| Gender | 0.426 | 0.655 | 0.109 | 0.52 | -0.877 | 1.728 |  |
| Age | 0.009 | 0.014 | 0.071 | 0.54 | -0.020 | 0.037 |  |
| Height | 0.056 | 0.041 | 0.235 | 0.18 | -0.025 | 0.137 |  |
| Weight | -0.008 | 0.020 | -0.043 | 0.71 | -0.048 | 0.032 |  |
| Adherence | -0.269 | 0.287 | -0.103 | 0.35 | -0.839 | 0.301 |  |
| Intervention | -1.154 | 0.375 | -0.327 | **0.003** | -1.899 | -0.408 |  |
