# Supplementary material for: Effect of Physician-Pharmacist Participation in the Management of Ambulatory Cancer Pain Through a Digital Health Platform: Randomized Controlled Trial
Source: JMIR Mhealth Uhealth. 2021 Aug 16;9(8):e24555. doi: 10.2196/24555 (PMC8406114; doi:10.2196/24555)
Supplement: Multimedia Appendix 10 [file mhealth_v9i8e24555_app10.doc]

**Multimedia Appendix 10.** The independent factors influencing least pain intensity.

| Parameters | β | SE | β’ | *P* - value | 95% CI of β | | R^2^ |
| --- | --- | --- | --- | --- | --- | --- | --- |
|  |  |  |  |  | Lower limit | Upper limit |  |
| Constant | -6.321 | 5.956 |  | 0.29 | -18.161 | 5.518 | 0.193 |
| Gender | 0.366 | 0.505 | 0.119 | 0.47 | -0.639 | 1.370 |  |
| Age | 0.003 | 0.011 | 0.035 | 0.76 | -0.019 | 0.025 |  |
| Height | 0.052 | 0.032 | 0.278 | 0.10 | -0.011 | 0.115 |  |
| Weight | -0.008 | 0.016 | -0.055 | 0.62 | -0.039 | 0.023 |  |
| Adherence | -0.525 | 0.221 | -0.256 | **0.02** | -0.964 | -0.085 |  |
| Intervention | -0.701 | 0.289 | -0.253 | **0.02** | -1.276 | -0.126 |  |
