# Supplementary material for: Effect of Physician-Pharmacist Participation in the Management of Ambulatory Cancer Pain Through a Digital Health Platform: Randomized Controlled Trial
Source: JMIR Mhealth Uhealth. 2021 Aug 16;9(8):e24555. doi: 10.2196/24555 (PMC8406114; doi:10.2196/24555)
Supplement: Multimedia Appendix 11 [file mhealth_v9i8e24555_app11.doc]

**Multimedia Appendix 11.** The independent factors influencing present pain intensity.

| Parameters | β | SE | β’ | *P* - value | 95% CI of β | | R^2^ |
| --- | --- | --- | --- | --- | --- | --- | --- |
|  |  |  |  |  | Lower limit | Upper limit |  |
| Constant | -9.149 | 8.887 |  | 0.31 | -26.821 | 8.523 | 0.095 |
| Gender | 1.078 | 0.761 | 0.251 | 0.16 | -0.436 | 2.592 |  |
| Age | 0.018 | 0.016 | 0.138 | 0.26 | -0.014 | 0.051 |  |
| Height | 0.063 | 0.047 | 0.243 | 0.18 | -0.030 | 0.157 |  |
| Weight | -0.017 | 0.023 | -0.087 | 0.46 | -0.063 | 0.029 |  |
| Adherence | -0.282 | 0.331 | -0.100 | 0.40 | -0.941 | 0.377 |  |
| Intervention | -0.598 | 0.432 | -0.155 | 0.17 | -1.456 | -0.261 |  |
